# Supplementary material for: Predicting visual function by interpreting a neuronal wiring diagram
Source: Nature. 2024 Oct 2;634(8032):113–23. doi: 10.1038/s41586-024-07953-5 (PMC11446822; doi:10.1038/s41586-024-07953-5)
Supplement: Supplementary file 6 — CRF and ERF predictions for individual TmY4 and TmY9 cells. Analogous to Supplementary Data 3, but for TmY target types. Shown are the top four monosynaptic pathways, the strongest pathway passing through each of the top ten intermediary types (ranking from Extended Data Fig. 7), and the trisynaptic pathway Tm1–TmY–Dm3–TmY (see the section entitled Prediction of spatial normalization). [file 41586_2024_7953_MOESM6_ESM.zip › DataS4/TmY9q/720575940638864454.pdf]

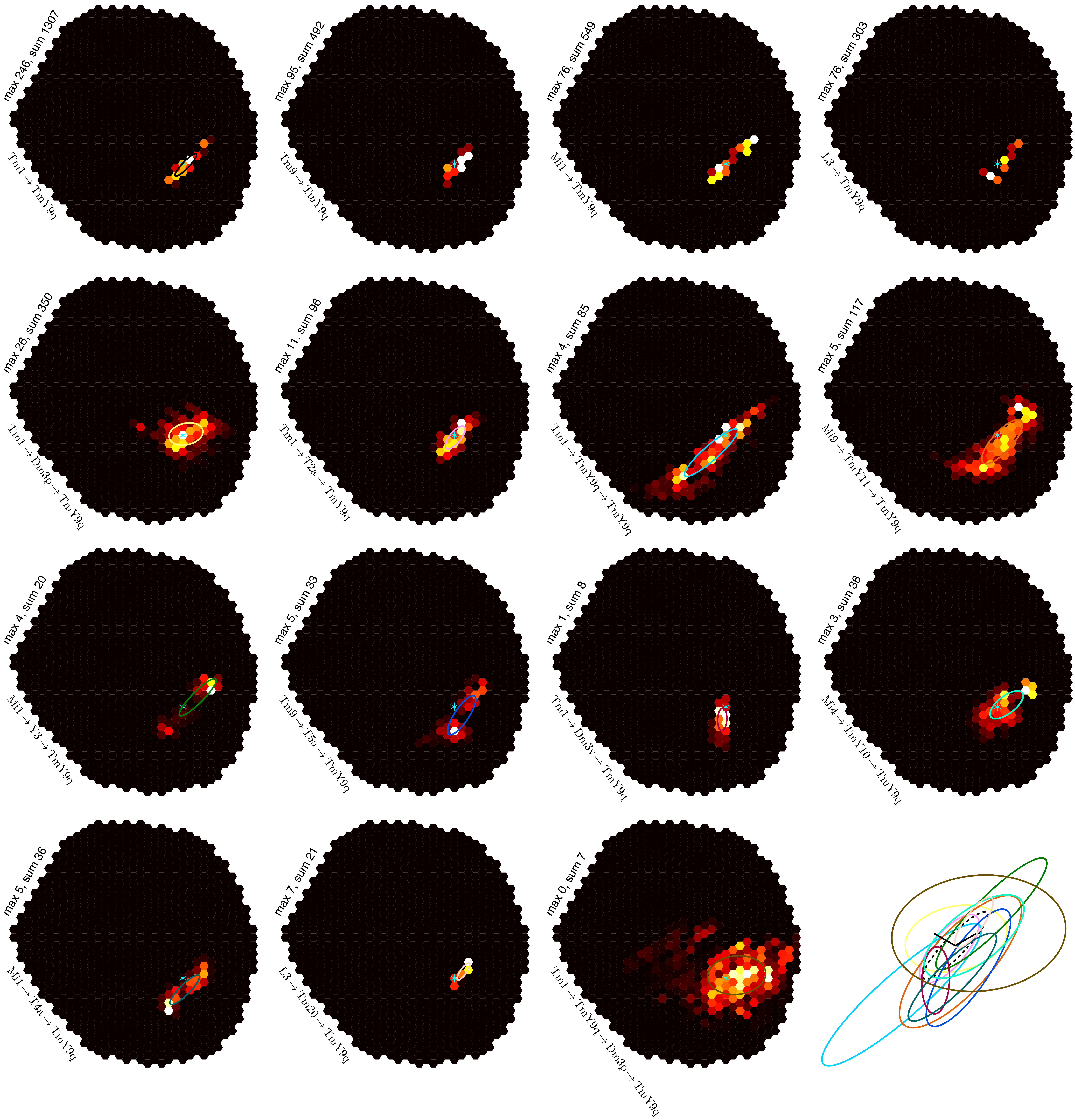
max 246, sum 1307
Tm1 → TmY9q
max 95, sum 492
Tm9 → TmY9q
max 76, sum 549
Mi1 → TmY9q
max 76, sum 303
L3 → TmY9q
max 26, sum 350
Tm1 → Dm3p → TmY9q
max 11, sum 96
Tm1 → T2a → TmY9q
max 4, sum 85
Tm1 → TmY9q → TmY9q
max 5, sum 117
Mi9 → TmY11 → TmY9q
max 4, sum 20
Mi1 → Y3 → TmY9q
max 5, sum 33
Tm9 → T5a → TmY9q
max 1, sum 8
Tm1 → Dm3v → TmY9q
max 3, sum 36
Mi4 → TmY10 → TmY9q
max 5, sum 36
Mi1 → T4a → TmY9q
max 7, sum 21
L3 → Tm20 → TmY9q
max 0, sum 7
Tm1 → TmY9q → Dm3p → TmY9q
